# Costs and effectiveness of a culturally tailored communication training program to increase cultural competence among multi-disciplinary care management teams

**DOI:** 10.1186/s12913-020-05662-z

**Published:** 2020-08-24

**Authors:** Marie Parker, Xiangming Fang, Andrew Bradlyn

**Affiliations:** 1 Kaiser Foundation Health Plan of Georgia, 3495 Piedmont Road, NE, Atlanta, GA 30305 USA; 2 Georgia State University School of Public Health, Urban Life Building, 140 Decatur Street, Suite 439, Atlanta, GA 30303 USA; 3 Center for Research and Evaluation, Kaiser Permanente, 1375 Peachtree Street, NE, Atlanta, GA 30309 USA; 4 China Agricultural University, 17 Qinghua E Rd, Haidian District, Beijing, China

**Keywords:** Cultural competence, Costs, Micro-costing, Cost-effectiveness

## Abstract

**Background:**

Several studies have demonstrated that cultural competence improves patient-provider communication, which promotes adherence to established care plans and improves patient satisfaction and health outcomes. However, there is very little data available regarding the costs associated with the development and implementation of cultural competence training, or the cost-effectiveness of these programs. To that end, this evaluation aims to describe costs, program effectiveness, and cost-effectiveness of a culturally tailored communication training program to improve cultural competence among multi-disciplinary care management teams.

**Methods:**

As part of a region-wide quality improvement initiative to reduce healthcare disparities among African American patients with uncontrolled hypertension, three multi-disciplinary care management teams were invited to participate in a two-part communication training program. A paired samples t-test was used to assess program effectiveness based on participant responses to a validated cultural competence self-assessment survey 2 weeks before and after the training program. A micro-costing approach was used to estimate programmatic costs for content development and delivery. Cost-effectiveness was then determined using the average cost-effectiveness ratio, and sensitivity analyses were conducted to assess the impact of participant mix on this result.

**Results:**

All scores (*n* = 17) improved after training; however, only the culturally competent behaviors (CCB) subscale change was statistically significant (*p* = 0.02). Overall program costs were $5754.19. The average program cost per participant was $138.51, with an ACER of $337.83 per 1-unit increase in CCB score. Sensitivity analyses yielded a range of ACERs between $122.59 and $457.07, where all participants are support staff or nurses, respectively.

**Conclusions:**

Culturally tailored communication training increases how frequently participants demonstrate culturally competent behaviors and may be a cost-effective intervention for care management teams to address individual cultural competence. Detailed costs associated with cultural competence training are largely unavailable in the literature; as such, these data may serve as a financial framework for organizations considering the implementation of similar programs.

## Background

In 2002, the Institute of Medicine released a groundbreaking report, Unequal Treatment: Confronting Racial and Ethnic Disparities in Health Care, that concluded racial and ethnic minorities in the United States receive lower quality of care and have higher rates of morbidity and mortality from chronic diseases than their white counterparts [1]. Nearly 20 years later, racial and ethnic disparities in healthcare access and quality of care have improved; yet, for nearly 19% of access measures and 75% of quality measures, the gap between Blacks and Whites persists today [2]. Moreover, the cost of racial disparities are significant, with estimated direct healthcare costs reaching $230 billion and indirect costs by more than $1 trillion [3].

Within the same groundbreaking report, healthcare provider training was identified as one of the most important tools to overcome disparities [1]. More often than not, the purpose of this training is to improve provider cultural competence. The concept of cultural competence first emerged in the late 1970s as cross-cultural healthcare and later, in the 1990s, expanded to include all racial and ethnic minority populations across the health system [4]. Many definitions of cultural competence exist, though most agree that culturally competent care respects the diversity of the patient and meets his/her social, cultural, and linguistic needs [5].

Several studies have tested the effectiveness of cultural competence training on intermediate outcomes, such as provider knowledge, attitudes, and behaviors, and long-term outcomes, like improvement in clinical measures; however, very few studies have reported detailed costs of cultural competence training [6, 7]. In one study where costs to train physicians were included, the overall cost for the course, excluding physician time, was provided but no cost-effectiveness analysis was conducted [8]. The current analysis goes beyond earlier evaluations to both describe costs associated with training and evaluate the cost-effectiveness of such a program aimed to improve cultural competence among multi-disciplinary care management teams.

## Methods

### Program design

As part of a region-wide quality improvement initiative to reduce healthcare disparities among African American patients with uncontrolled hypertension, a two-part culturally tailored communication training program was developed and delivered to care management teams.

### Participants

Three care management teams, comprised of 14 registered nurses, 15 registered pharmacists, and 7 non-clinical support staff, were invited to participate in the training program and self-assessment. Prior to the initiation of the program, all participants were informed that their participation in the program and assessment was voluntary.

### Training program

The training was adapted from a series of resources and similar quality improvement initiative developed by, and with, Kaiser Permanente’s Equitable Care Health Outcomes team that incorporated an evidence-based communication model (AIDET – Acknowledge, Introduce, Duration, Explanation, and Thank You) and general awareness of African American culture and barriers within the healthcare system [9]. The newly developed training program consisted of a 25-min on-demand web-based module and 1.5 h live interactive session. The web-based module, “Touching the Dream: Focus on African American Culture and Health”, is part of the Diversity & Health Series created in the Kaiser Permanente Colorado Region to raise cultural awareness of healthcare providers to eliminate healthcare disparities within African American communities. The live interactive session included the delivery of a presentation, “Building Connections Using Culturally Tailored Communication: AIDET Training for African American/Black Patients”, and participant discussion of scenarios and experiences.

### Effectiveness outcomes

The primary effectiveness outcome was estimated using a paired samples t-test based on the change in self-assessed cultural competency from baseline on one index and two scales within the Cultural Competence Assessment (CCA) instrument before and after the training program. The CCA was developed to assess cultural competency in a multi-disciplinary setting, with adequate test-retest validity overall (*r* = 0.85, *p* = 0.002) and for each of the subscales, culturally competent behaviors (CCB, *r* = 0.87, *p* = 0.002;) and cultural awareness and sensitivity (CAS, *r* = 0.82, *p* = 0.002) and acceptable reliability (Cronbach’s alpha = 0.89) [10, 11]. The instrument was used with permission from its creators. The index item assesses overall self-reported cultural competence using a single 5-point Likert-like question, “Overall, how competent do you feel working with people who are from other cultures different from your own?” The CAS subscale uses a 7-point Likert-like response set ranging from “strongly agree” to “strongly disagree”, while the CCB subscale uses a similar 7-point scale with responses from ‘always’ to ‘never’. Responses are translated to numerical values and summed to yield a score for the CCA index and both the CAS and CCB subscales, where higher scores reflect greater self-reported overall competence, greater knowledge, more positive attitudes, and more frequently demonstrated behaviors.

### Program costs

Program costs were calculated using a micro-costing approach, where resource units are multiplied by unit costs. All participants were employees of Kaiser Foundation Health Plan at the time of training. Administrative time to develop and deliver the intervention was tracked throughout the program. Participant salaries and fringes were estimated using the most recent Bureau of Labor median occupation wages for the Atlanta-Sandy Springs-Roswell, GA metropolitan service area for the state of Georgia and employer costs for employee compensation, respectively [12, 13]. For those participants that were not located at the regional office where the training took place, travel time and distance for participants were estimated based on average time and distance from medical offices to the regional office and mileage reimbursement was calculated based on the January 1, 2019 standard mileage rate as dictated by the Internal Revenue Service [14].

### Cost-effectiveness analysis

The cost-effectiveness analysis was from the employer perspective. Mean intervention costs (the costs associated with the training program) and mean effectiveness gained (the difference between pre- and post-training CCA scores, where statistically significantly different) were used to calculate the average cost-effectiveness ratio (ACER)(Eq.1). The ACER was used for this analysis because it is often used by organizations as a summary parameter that characterizes an intervention independent of a comparator, which this new program lacked, beyond the status quo, to systematically allocate budgetary resources [15, 16].

1 $$ ACER=\frac{ProgramDeliveryCost}{AverageScoreAfterTraining- AverageScoreBeforeTraining} $$

Sensitivity analysis was applied to establish how the costs and cost-effectiveness of the program might vary from the base-case for three participants mix scenarios: all nurses, all pharmacists, or all support staff.

Institutional Review Board review was not sought for this work, as the authors determined the project did not meet the Federal definition for human subjects research, instead, falling within the scope of quality improvement. Quality improvement initiatives are exempt from Institutional Review Board review, by definition, as these initiatives do not aim to create new knowledge, per se, but rather use existing knowledge to improve health outcomes within a local system [17, 18].

## Results

Of those invited to participate in the training program, 92% (*n* = 34) completed the web-based module and attended the group session. There were no observed differences between respondents and non-respondents.

### Effectiveness

Survey responses were received from 100% (*n* = 36) of participants before the training and 61% (*n* = 22) after the training. Of the participants that completed the self-assessment before and after the training, 47% (*n* = 17) disclosed enough information to match responses for inferential analysis (Table 1).

**Table 1:** Mean Cultural Competence Assessment (CCA) Scores (Matched) Before and After Training (*n* = 17)

	Before Training	After Training	*p*-value
Overall Cultural Competence, mean (SD)	4.59 (0.51)	4.76 (0.43)	0.09
Cultural Awareness and Sensitivity, mean (SD)	6.03 (0.41)	6.04 (0.48)	0.46
Culturally Competent Behaviors, mean (SD)	4.31 (0.90)	4.72 (0.93)	0.02

There was no statistically significant difference between overall cultural competence scores before (*M* = 4.59; *SD* = 0.51) and after training (*M* = 4.76; *SD* = 0.43); t (16)=1.74; *p* = 0.09. Similarly, no statistically significant difference was observed between CAS scores before (*M* = 6.03; *SD* = 0.41) and after training (*M* = 6.04; *SD* = 0.48); t (16)=1.74; 0.46. However, there was a statistically significant difference between CCB scores before (*M* = 4.31; *SD* = 0.90) and after training (*M* = 4.72; *SD* = 0.93); t (16)=1.74; *p* = 0.02.

### Costs

Costs were divided into two categories: program development and program delivery. The costs to develop the training program, $1044.98, were predominately administrative while costs to deliver the program, $4709.21, were largely attributed to participant time (Table 2).

**Table 2:** Total Costs to Develop and Deliver the Culturally Tailored Communication Training Program

Type of Cost	Resource Unit	Unit Cost ($)^a^	Total Cost ($)
Program Development (One-Time Costs)			
Time for planning meetings	5 h	$55.65 (lead) $19.65 (HR consultant) $12 (intern)	$566.58
Time to modify existing presentation	2 h	$55.65 (lead)	$144.47
Fee to print training materials	36 handouts	$1.25 per handout	$45
Time for facilitator training	4 h	$55.65 (lead)	$288.93
Total			$1044.98
Program Delivery (Fixed and Variable Costs)			
Time to complete web-based module	28 min	$56.09 (pharmacist) $33.92 (nurse) $19.90 (support staff)	$813.72
Time to setup	30 min	$55.65 (lead) $19.65 (HR consultant) $12 (intern)	$56.66
Time to facilitate training	1.5 h	$55.65 (lead)	$108.36
Time to travel to training^b^ (average time from medical offices to regional office)	30 min	$56.09 (pharmacist) $33.92 (nurse)	$781.43
Time to attend training	1.5 h	$56.09 (pharmacist) $33.92 (nurse) $19.90 (support staff)	$2615.54
Miles from worksite to training siteb (average distance from medical offices to regional office)	25 miles	$0.58/mile (off-site attendees)	$333.50
Total			$4709.21

^a^Fringe benefits of 29.8%, from the Bureau of Labor, were included in all wage calculations

^b^Some nurses and all support staff were located at the regional office

### Cost-effectiveness

Base-case and sensitivity analysis costs and ACERs are reported in Table 3. The base-case ACER was $337.83 per 1-unit increase in the frequency with which care management teams demonstrate culturally competent behavior. The sensitivity analyses yielded a range of ACERs for a 1-unit change in CCB performance between $122.59 when all participants are support staff to $457.07 when all participants are nurses.

**Table 3:** Base-case and Sensitivity Analysis Intervention Costs and Cost-effectiveness Ratios^a^

Variable	Base-case	Nurse-only	Pharmacist-only	Support staff-only
Mean intervention costs, $	$138.51	$123.41	$194.49	$68.65
Mean effectiveness gained^b^	0.41	0.27	0.83	0.56
ACER, $ per 1-unit increase in frequency of culturally competent behaviors (CCB)	$337.83	$457.07	$234.32	$122.59

*Abbreviations*: *ACER* average cost-effectiveness ratio, *CCB* culturally competent behavior subscale of cultural competence self-assessment

^a^Values account for program delivery costs listed in Table 2

^b^Mean effectiveness gained is from the matched sample (*n* = 17) and uses the CCB subscale, as this was the only statistically significantly effectiveness outcome

## Discussion

Culturally tailored communication training significantly improved the frequency with which members of care management teams within an integrated delivery network reported demonstrating culturally competent behaviors. This finding is consistent with those of other larger, more robust interventions that reported beneficial effects of cultural competence training on healthcare providers’ skills and behaviors [6]. Of the three dimensions assessed within the cultural competence assessment, only culturally competent behaviors significantly increased, likely because the training program provided communication tools that could be implemented in every day practice. This finding is encouraging as communication is one behavior which cultural competence training programs aim to improve among healthcare providers and teams.

No significant improvement in overall cultural competence or cultural awareness and sensitivity was found after our training program. Participants’ self-assessments on each of these cultural competence measures was high prior to the training, which may be explained in that 75% of survey respondents indicated they had received prior cultural competence training. This explanation is consistent with the contrasted groups assessment within the CCA psychometric evaluation study, where scores for those that reported receiving training previously were significantly higher than those without prior training [11]. Moreover, Kaiser Permanente has long been recognized for its organizational climate that fosters cultural competence, which a recent study found has a direct impact on providers’ confidence in their own cultural competence [19].

The total cost to develop and deliver the training was approximately $5800; however, nearly 20% reflects a one-time cost to the organization for the development of the initial program. It is possible that these costs may underestimate the costs to other organizations for the development of a similar training program as there were no costs incurred for the “Touching the Dream” video, initial training content (which was modified), and rental space. Even so, the Office of Minority Health offers many educational resources at no charge through its website, Think Cultural Health, which organizations may use to improve cultural competence across many different healthcare roles [20]. To minimize the disclosure of sensitive financial information, local average wages and fringes from labor bureau statistics were used in this analysis, so organizations may choose to compare these data to actual salaries and wages before moving forward with developing their own cultural competence training program.

Because this was a new program and there were no active programs to which a comparison could be made, the cost-effectiveness analysis used for this program evaluation was the ACER. The use of an ACER to report the results of a CEA are likely less well known to the reader than the use of incremental cost effectiveness ratios (ICERs). However, well-known organizations have recommended the use of ACERs for similar purpose and the evaluation of de novo interventions, e.g. the World Health Organization recommends using ACERs for programs where resource allocation is limited by budgetary constraints and the Centers for Disease Control and Prevention has published an ACER-based CEA of West Nile vaccination [16, 21].

The average cost of the program per participant was $138.51. The ACER of the training program was $337.83 per 1-unit increase in program effectiveness. Program effectiveness used in the calculation of the ACER was based solely on the culturally competent behavior (CCB) subscale, as this was the only statistically significant improvement observed in our analysis. Most cost-effectiveness analyses (CEAs) use quality-adjusted life years (QALYs) with commonly agreed upon thresholds upon which cost-effectiveness is compared. There are few thresholds available for CEAs based on natural units; instead, the costs of the program are often compared to the costs of an occurred or avoided event. There is no readily available data regarding healthcare costs associated with the frequency of culturally competent behaviors which leaves organizations to subjectively assess the worth of increasing the frequency of nurses, pharmacists, and non-clinical support staff from “often” to “somewhat often” or “very often” to “always”.

When participant role and effectiveness by role was altered, the ACERs ranged from $122.59 (support staff only) to $457.07 (nurses only). These ACERs underscore the impact of balancing financial investment in the training program and the expected outcome of the program. For instance, pharmacists reported the largest improvement from baseline (0.83) yet their salaries tend to be roughly 65% higher than nurses and nearly triple that of support staff, which yielded an ACER one-half that of the nurses-only scenario. The lowest ACER, $122.59, represented the support-staff only scenario and may present the greatest return on the organization’s investment as some case studies have demonstrated the role that these frontline individuals can play in improved clinical and financial outcomes [22].

There are several limitations of this evaluation. The small sample size significantly limits our ability to confidently demonstrate an effect on cultural competence because of the training. However, it is likely that other programs would implement this type of training in groups of similar size in accordance with standards set forth by accrediting bodies like The Joint Commission or professional associations like the American Nurses Association [23, 24]. Therefore, the value of this analysis may lie more in the micro-costing data than the methodological limitations of the effectiveness analysis. While we accounted for the lack of an active comparator by using an appropriate CEA method, future research should seek to evaluate the differences between various training programs that would likely result through iterations of quality improvement work within an organization. We acknowledge that, given the organizational culture, employees’ self-assessed cultural competence may not represent that of other organizations; employees or those in other healthcare settings. There is no information on sustained changes in cultural competence as changes in self-reported cultural competence, cultural awareness and sensitivity, and culturally competent behaviors were only assessed at one point in time, 2 weeks following the initial training. Participants’ responses may have been biased by concerns about social desirability or perceived social norms. Lastly, program effectiveness was limited to participant self-report and did not consider other outcomes like patient experience or improvement in clinical parameters.

## Conclusions

Despite these limitations, we believe this paper provides substantive contributions to the vast body of cultural competence training outcomes. The outcome of the program is congruent with other programs that aim to improve skills and behaviors. Moreover, this is the first cultural competence training evaluation that includes micro-costing and cost-effectiveness outcomes, which may serve as a financial framework for other organizations considering the implementation of a similar program.

## Data Availability

The datasets generated and/or analyzed for this evaluation are not publicly available due to the sensitive nature of employee and employer data.

## Abbreviations

ACER: Average cost-effectiveness ration
AIDET: Acknowledge, Introduce, Duration, Explain, Thank
CAS: Cultural awareness and sensitivity
CCA: Cultural competence assessment
CCB: Culturally competent behaviors
CEA: Cost-effectiveness analysis
QALY: Quality-adjusted life years
